# Trends in the Use of Botanicals in Anti-Aging Cosmetics

**DOI:** 10.3390/molecules26123584

**Published:** 2021-06-11

**Authors:** Marta Salvador Ferreira, Maria Catarina Magalhães, Rita Oliveira, José Manuel Sousa-Lobo, Isabel Filipa Almeida

**Affiliations:** 1 Laboratory of Pharmaceutical Technology, Department of Drug Sciences, Faculty of Pharmacy, University of Porto, Rua Jorge de Viterbo Ferreira, 228, 4050-313 Porto, Portugal; msbferreira@ff.up.pt (M.S.F.); maria.c.magalhaes@cuf.pt (M.C.M.); slobo@ff.up.pt (J.M.S.-L.); 2 UCIBIO/REQUIMTE, MedTech, Laboratory of Pharmaceutical Technology, Department of Drug Sciences, Faculty of Pharmacy, University of Porto, Rua Jorge de Viterbo Ferreira, 228, 4050-313 Porto, Portugal; 3 Biomedical Research Centre (CEBIMED)/Research Centre of the Fernando Pessoa Energy, Environment and Health Research Unit (FP-ENAS), Faculty of Health Sciences, University of Fernando Pessoa, 4249-004 Porto, Portugal; ritao@ufp.edu.pt

**Keywords:** botanical, preparations, anti-aging, cosmetics, market

## Abstract

Botanical ingredients have been used for thousands of years in skincare for their convenience as well as the diversity and abundance in compounds with biological activity. Among these, polyphenols and especially flavonoids have gained increasing prominence due to their antioxidant and anti-inflammatory properties. In this study, the most used botanical preparations in anti-aging products marketed in 2011 were determined. The analysis was repeated in 2018 for new and reformulated products. The scientific evidence for their application as active ingredients in anti-aging cosmetics and their flavonoid content was also compiled by searching in online scientific databases. Overall, in 2018, there was a noticeable increase in the use of botanical preparations in anti-aging cosmetics. However, the top three botanical species in both years were *Vitis vinifera*, *Butyrospermum parkii,* and *Glycine soja*, which is consistent with the greater amount of scientific evidence supporting their efficacy. Regarding the function of botanical preparations, there is a clear preference for DNA-protecting ingredients. The most prevalent flavonoids were flavan-3-ols, proanthocyanidins, and anthocyanins. This study provided an updated overview of the market trends regarding the use of botanicals in anti-aging products and documented the state of the art of scientific evidence for the most used plants.

## 1. Introduction

For thousands of years, naturally derived ingredients have been used as raw materials of skin care products, being derived from mineral, animal, or vegetable sources [1,2].

In the 21st century, the use of naturally derived ingredients is still a growing trend, which is possibly due to the influence of the internet and social media. From 2015 to 2019, the global “natural cosmetics” market has been expanding, with 10–11% annual growth. This market also represents a great opportunity for the cosmetic industry, since many consumers are willing to pay more for these products [3,4]. 

In 2011, approximately one-third of ingredients listed by the International Nomenclature of Cosmetic Ingredients (INCI) system at the Personal Care Products Council were classified as “botanical extracts”. Botanical ingredients may result from different processing methodologies of the same plant material, including plant extracts, expressed juices, tinctures, waxes, vegetable oils, lipids, plant carbohydrates, essential oils, as well as purified plant components, such as vitamins, antioxidants, and other substances with recognized biological activity [5]. The INCI name uses the Latin binomial indicating the part of the plant (e.g., root, leaf), and the extraction product (e.g., extract, oil, juice). It is noteworthy that not all these parameters are always indicated in the label of cosmetic products [6].

Of all the components that can be found in botanical preparations for cosmetic use, polyphenols have gained increasing prominence due to the plethora of biological activities. Polyphenols were found to provide antioxidant and anti-inflammatory activity after topical application, as well as an ability to inhibit the gene expression and activity of skin enzymes, such as hyaluronidase, matrix metalloproteinase (MMP) collagenase, and serine protease elastase [7]. 

Polyphenols are a large group of natural, synthetic, and semi-synthetic compounds, with at least one phenolic ring. Polyphenols are separated in some classes and various subclasses depending on the number of aromatic rings, namely phenolic acids, including hydroxybenzoic and cinnamic acids, flavonoids, and stilbenes, amongst others [8]. Flavonoids are the major group of low molecular weight phenolic compounds and have the general structure of a 15-carbon skeleton, which consists of two phenyl rings (A and B) and a heterocyclic ring (C), comprising a large family that includes flavanols, flavonols, flavones, anthocyanidins, and isoflavones, amid others [9].

Parallel to the “natural” segment, the whole cosmetics market has been growing with the “anti-aging” segment holding a share of over 39.6% in 2015 [10]. Skin aging is an inevitable result from the cumulative consequences of a cell’s chronological aging, but it is also exacerbated with the exposure to multiple environmental factors known as the skin aging exposome. These include radiations (ultraviolet, visible, and infrared), air pollution, tobacco smoke, poor nutrition, as well as sleep deprivation, stress, or the inadequate use of cosmetics [11]. The exposure to light sources such as sun and artificial light seems to be particularly relevant, leading to a phenomenon called photoaging (Table 1) [12]. Blue light from the sun and electronic devices, also known as high energy visible light, is being proposed as an important factor for skin aging, especially regarding pigmentation [11]. The causes and consequences associated with chronologically and photoaged skin are summarized in Table 1. 

In 2010, a study assessed the top 10 botanical ingredients in over-the-counter anti-aging creams at the United States of America. We are unaware of any similar work addressing any European cosmetics’ market [13].

Herein, this study reports the most commonly used botanical species in anti-aging cosmetics marketed in 2011 and 2018. A critical appraisal of their composition and the current scientific evidence that supports their anti-aging efficacy was also performed.

**Table 1:** Causes and consequences in chronologically and photoaged skin.

	Chronologically Aged Skin	Photoaged Skin
**Leading** **Factors and Mechanisms**	Reactive oxygen species (ROS) Generated during oxidative cell metabolism, mostly at mitochondria [14,15]; DNA damage Telomer shortening, gene loss, decreased DNA methylation and phosphorylation reaction, decreased DNA repair, oncogene and tumor suppressor gene deregulation [16,17]. Hormonal changes Estrogen decline during menopause, testosterone decline both in men and women [18,19].	UVB (short wavelength) light UVA (long wavelength) light Their energy is transferred to generate ROS, causing transcription factor activation, lipid peroxidation, (MMP-1 expression, and DNA-strand breaks [15,20] Visible light (especially blue light) That induces ROS, MMP-1 and IL-1 and deploys skin carotenoids [11] Infrared light Produces heat and increases MMP-1 production [11].
**Histological Findings**	Epidermis [15] thinning; Flattening from dermal–epidermal junction. Dermis [15,20] General atrophy of the extracellular matrix; Reduced collagen production.	Epidermis [15] Stratum corneum compaction; Increased thickness of the granular layer; Epidermal thickness may be either reduced or increased; Dermal–epidermal junction atrophy; Increased number of hypertrophic melanocytes; Dermis [15,20,21] Mature collagen fibrils are degenerated and replaced by disorganized and fragmented collagen (basophilic degeneration); Elastin increase, occupying areas previously inhabited by collagen fibers (solar elastosis).
**Clinical Findings**	Fine wrinkles; Laxity; Xerosis; Loss of firmness; Benign neoplasms such as seborrheic keratosis and cherry angioma [14,15].	Coarse wrinkling; Roughness; Hyperpigmentation; Inelasticity; Superficial vascular abnormalities; Pre-malignant actinic keratosis. [14,15]

## 2. Results and Discussion

### 2.1. Botanical Preparations Prevalence and Variety

In 2011, 63.8% of anti-aging products contained botanical preparations while in 2018, 73.8% of products contained these ingredients. This corresponds to a 16% increase in a seven-year period, which is consistent with market growth trends [3].

The number of botanical species used in anti-aging cosmetic products per year was slightly higher in 2011, with 106 different species versus 96 in 2018. However, 177 products were analyzed in 2011 comparing with 103 products in 2018, which could have influenced this finding.

### 2.2. Top Botanical Species

**Figure 1:**
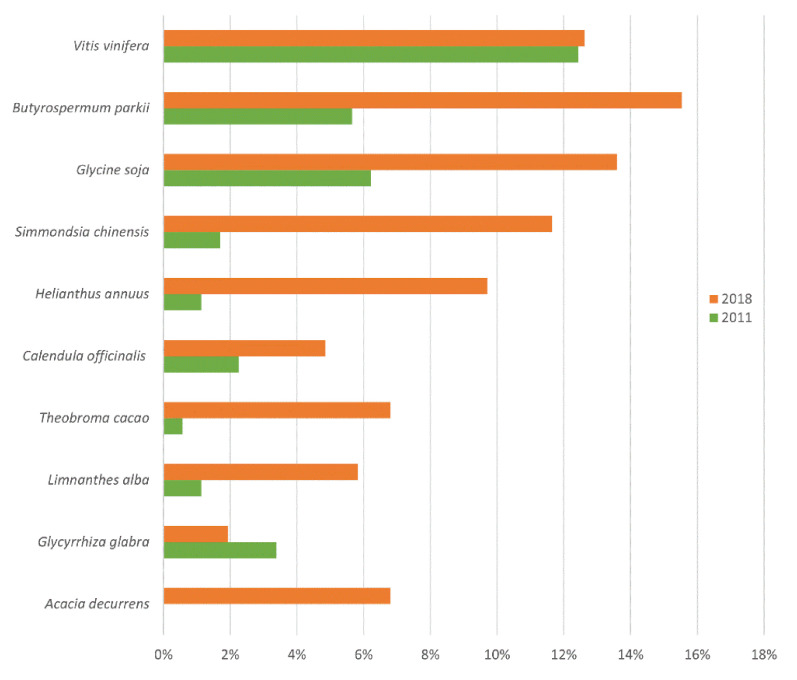
Top botanical species included in the composition of anti-aging products marketed in 2011 and 2018.

The ten botanical species with greater prevalence are presented in Figure 1.

However, there are many different preparations for some botanical species, corresponding to the extraction of different parts of the plant and different extraction methods. Aside from the variables regarding the plant origin, these differences alone can lead to very diverse ingredients. Additionally, in some cases, the information found in the products’ composition list is incomplete, and it does not allow identifying which part of the plant or extraction method were used. The information presented in the cosmetic product label was compiled regarding each botanical preparation and then categorized according to the botanical species (Table 2).

It was observed that nine of the ten most used botanical species occurred both in 2011 and 2018 (Figure 1), which suggests these play an important role in the efficacy of the cosmetic product. It is also worth mentioning that aside from *Glycyrrhiza glabra*, there was an increase in the use of the Top 10 plant species in 2018 compared to 2011. This finding is consistent with our results regarding botanical preparations prevalence. Below, the scientific evidence supporting anti-aging efficacy regarding all botanical species from the top 10 is reported. The polyphenol composition from all botanical preparation is summarized in Table 3.

#### 2.2.1. *Vitis vinifera*

In 2011, *Vitis vinifera* (vine) was the most used botanical species, turning to the third place in 2018. From all the botanicals analyzed in this study, it is the one presenting the largest variety of preparations. 

Grape and red wine are among the major dietary sources of stilbenes both in edible and non-edible plant tissues [22].

“Palmitoyl grapevine shoot extract” was the most used grape preparation in both years, although its use has decreased from 2011 to 2018. The composition of this palmitoyl extract is unknown. However, after vine stems, shoots are the part of the plant that contain a greater resveratrol concentration [23]. Cis- and trans-resveratrol are abundant polyphenols in the aerial parts from the plant. They provide antioxidant activity and downregulate the expression and activity of ROS generating enzymes while increasing the expression of antioxidant enzymes. Resveratrol has shown to control metalloproteinase-1 (MMP-1)-mediated UVB-induced skin aging, apoptosis-induced skin aging, and inflammation-mediated complications called “inflammaging” in dermal fibroblasts [24]. The topical application of resveratrol to SKH-1 hairless mice before UVB exposure also resulted in a significant inhibition of skin edema, inflammation, and lipid peroxidation [25]. Grapevine shoot extracts are also known to contain multiple stilbenoids, such as *trans*-resveratrol, ampelopsin A, ε-viniferin, r-viniferin, ω-viniferin, pallidol, hopheaphenol, piceatannol, isohopeaphenol, and r2-viniferin [26]. Trans-ε-Viniferin, a resveratrol oligomer, was shown to provide a greater tyrosinase inhibition effect when compared with resveratrol, arbutin, kojic, and ascorbic acids [27]. An in vitro study determined that grape shoot extract appeared to have significantly stronger antioxidant action than vitamin C or vitamin E on keratinocytes after H_2_O_2_ exposure [28]. An in vivo evaluation showed that a four-week twice-daily application of 1% *Vitis vinifera* shoot extract (also known as sarmentine) serum provided a significant improvement in skin firmness, radiance, texture, fine lines, and wrinkles [29].

From 2011 to 2018, the use of “*Vitis vinifera* (grape) seed oil” has decreased, while “Palmitoyl grape seed extract” and “*Vitis vinifera* seed extract” were only used in the later years (Table 2). Grape seed oil contains mainly linoleic acid in its fatty acid composition, which composes 66.0% to 75.3% of the total fatty acid amount. It also contains a higher vitamin E content than soybean and olive oils, which together with phenolic compounds such as catechins, epicatechins (flavan-3-ols), procyanidin B1 (proanthocyanidin) flavonoids, carotenoids, phenolic acids, and stilbenes provides an antioxidant activity that may be useful in anti-aging cosmetics. Grape seed oil is used as an emollient in cosmetic products. It also has been shown to provide additional benefits to the skin such as antimicrobial activity and wound-healing promotion in rat models [30]. However, there is still lacking scientific evidence in that regard. Grape seed extracts are especially rich in proanthocyanidins, mainly B type procyanidins but also monomers and oligomers, which have been shown to be potent antioxidants and free radical scavengers, being more effective than either vitamin C or vitamin E. Grape seed extracts also contain catechin, epicatechin, and epicatechin gallate [13,31]. These preparations have shown a tyrosinase-inhibiting activity, being useful in anti-aging cosmetics [32]. A clinical study evaluated the effects in human facial skin of a W/O cream containing a Muscat Hamburg black grape seed extract. This single-blinded randomized placebo-controlled study showed a significant result for skin whitening, moisturizing, and potential anti-aging effects [33]. The greater amount of evidence from the seed extracts compared to the oil may justify their increasing use [25,34]. In fact, “*Vitis vinifera* seed extract” has been proposed as a cosmeceutical active ingredient and anti-pollution ingredient [13,35]. Nevertheless, the exact composition from the “Palmitoyl grape seed extract” remains unknown.

“*Vitis vinifera* (grape) fruit extract” was also used in 2011, but it has not been found in 2018. Grape berries contain multiple antioxidants, such as vitamins C, E, carotenoids, and polyphenols [36]. In fact, they are considered as one of the most important dietary fruit sources of bioactive polyphenols such as anthocyanins, flavonols, flavan-3-ols, tannins, hydroxycinnamic acid derivatives, and stilbenes, such as resveratrol [28,37]. A large amount of these compounds is present in grape skin (especially in red-skinned varieties), seeds, and, to a lesser extent, in pulp [37]. Vitamin C (ascorbic acid) is well known for its anti-aging effects on skin, improving its resistance to UV exposure, minimizing hyperpigmentation, reducing wrinkle scores, and improving skin texture [38,39]. Vitamin E (tocopherol) is also used as anti-aging active ingredient due to its ability to reduce erythema caused by UV exposure, roughness, sunburn, wrinkling, and skin pigmentation [15]. Melatonin was also found in berry skin from Italian and French grapes. This neurohormone is a biogenic indolamine that performs an important role in the regulation of circadian and seasonal rhythms, but it is also a proven free radical scavenger and broad-spectrum antioxidant. Contrary to vitamins C, E, or glutathione, which can be regenerated by redox reactions and may promote the formation of other oxidized species, melatonin seems to interact with free radicals by addition reactions, thus resulting in stable products that are antioxidants themselves [40]. A randomized, placebo-controlled, double-blind study showed that the topical application of melatonin provides a protective effect against erythema induced by UV radiation from natural sunlight [41]. The clinical efficacy of topical melatonin as an anti-aging active ingredient remains unknown. However, a study comparing two day and night formulations containing melatonin with a non-treated control side showed an improvement in skin hydration and skin tonicity, with a clinical improvement in the aspect of wrinkles, as the instrumental results were not significant compared with baseline and control sides [42]. Although the grape juice may have a promising composition, studies demonstrating its efficacy for combating skin aging were not found. This lack of evidence could explain its reduced application in cosmetic products.

#### 2.2.2. *Butyrospermum parkii*

*Butyrospermum parkii* (shea, or *Vitellaria paradoxa*) use has increased from 2011 to 2018, taking the first place as the most used botanical (Figure 1). Shea is mainly used for its butter, which consists of a solid fat extracted from mature shea fruit. 

It contains 90% triglycerides (saponifiable fraction) and 10% non-triglycerides (unsaponifiable fraction). The main fatty acids found in shea are stearic, oleic, palmitic, linoleic, and arachidic acid, which provide moisturizing and barrier protective actions [43,44]. The unsaponifiables include antioxidants (oil soluble tocopherols), triterpenes (e.g., butyrospermol), phenols, sterols, karitene, allantoin, and polyphenols (mainly the catechin), which together have been shown to provide UV-B absorbing properties [45,46]. Shea butter has shown to boost collagen production while inactivating proteases such as metalloprotease (e.g., collagenase) as well as serine protease (e.g., elastase) [45]. Two clinical studies showed that shea butter is able to reduce multiple signs of aging and prevent photo-aging [47]. 

Aside from “*Butyrospermum parkii* (shea) butter”, whose use almost tripled in 2018, one product containing “*Butyrospermum parkii* (shea) Butter Extract” was also found in 2018, which contains a higher bioactive fraction of shea butter triterpene esters [48]. The evidence regarding shea butter advantages for skin aging may justify the increased use in anti-aging products from 2011 to 2018 as well as the development of differentiated preparations.

#### 2.2.3. *Glycine soja*

In 2018, *Glycine soja* (soy) was the second most used botanical, with many parts of the plant being used in cosmetic products (Figure 1). Soy (*Glycine max* L.) belongs to the Fabaceae pea family, and it is native to southeastern Asia. It has been used in traditional Chinese, and it started being planted in the US during World War II [49]. 

“*Glycine soja* (soybean) oil” provides moisturizing and lubricating effects to skin care products. Its composition consists of triglycerides of linoleic (54%), oleic (24%), and linolenic (7%), and saturated fatty acids [50]. Soybean oil had a 6-fold increase in anti-aging products from 2011 to 2018, although no studies in the scientific literature demonstrate an anti-aging action. 

Protein is the major constituent of the soybean (30 to 50 g/100 g), with β-conglycinin (7S) and glycinin (11S) representing 65% to 80% of total protein amount. Whole soybean contains about 7 to 9% of protease inhibitors, mainly STI (Kunitz-type trypsin inhibitor) and soybean trypsin BBI (Bowman–Birk protease inhibitor) [51]. Non-denatured soybean preparations, containing STI and BBI, result in skin lightening demonstrated both in vitro and in vivo by reducing melanosomes phagocytosis thus preventing melanin transfer from keratinocytes [51,52]. 

Soybean germ is the seed fraction with higher content of antioxidant and antiproliferative phytochemicals, such as soyasaponins, tocopherols, and phytosterols. The germ may be 6 to 10-fold more concentrated in total isoflavones than cotyledons (embryonic leaves) [53]. There is a pending patent regarding the use of soybean germ extract in combination with creatine, creatinine, or derivatives for stimulating collagen synthesis and reducing the signs of aging. However, no evidence of this action was found [54]. Soybean germ also contains relevant flavonoids such as genistein, equol, and daidzein isoflavones, which provide antioxidant, anti-inflammatory, and estrogenic effects, as well as estrogenic lignans [15,51]. Isoflavones, and especially genistein, also provide a photoprotective effect. Soybean extracts showed to inhibit elastases while increasing skin elastin, collagen synthesis, and the glycosaminoglycans levels, especially hyaluronic acid (HA) in aging skin [49,51,55]. These effects have shown to provide a positive impact on photoaging in four double-blind controlled trials, which were performed with whole soy, soy milk, and soy isoflavones plus lignans [15]. Soy milk, soybean, and soy isoflavones, especially genistein, have been proposed as cosmetic active ingredients [35,56]. “Soy isoflavones” and “*Glycine soja* (soybean) germ extract” were only used in 2018, which was possibly due to their anti-aging benefits. Although soy germ extract also contains isoflavones, its application as an anti-aging active ingredient is still inconclusive.

“Hydrolyzed soy protein”, which provides small peptides and isolated amino acids to the skin was only used in 2011. Its benefits in anti-aging cosmetics are not documented to this day.

#### 2.2.4. *Simmondsia chinensis*

From 2011 to 2018, the use of *Simmondsia chinensis* (jojoba, or Buxus chinensis) also increased. Jojoba belongs to the Buxaceae family, and its oil is widely used in in cosmetic formulas [32,57]. It contains a broad spectrum of fatty acids such as oleic, linoleic, linolenic, and arachidonic as well as triglycerides, which together have a composition that is similar to skin sebum [32]. Jojoba oil also provides an antioxidant activity due to its content in polyphenols such as tannins, as well as alkaloids, steroids, and glycosides [58]. The chemical identity of these compounds is not known.

“*Simmondsia chinensis* (jojoba) seed oil”, found in 2011, seems to have been replaced by “*Simmondsia chinensis* oil”, which probably corresponds to the same preparation. 

Moreover, in 2018, we have also found “Jojoba esters” in several products. However, their special interest for the application on anti-aging cosmetics is unknown.

#### 2.2.5. *Helianthus annuus*

*Helianthus annuus* (sunflower) is also used in cosmetics for the fatty content of the seeds. Sunflower seed oil consists mainly of oleic and linoleic acids, presenting a higher concentration from the latter when compared to olive oil [57]. Linoleic acid is an agonist of the peroxisome proliferator-activated receptor-alpha (PPAR-α), which enhances keratinocyte proliferation and lipid synthesis. Thereby, the linoleic acid content is hypothesized as the main reason why sunflower oil has been shown to preserve stratum corneum integrity and improve hydration of the adult skin without inducing erythema [59]. Sunflower oil also contains polyphenols such as caffeic, chlorogenic, and ferulic acids [60]. “*Helianthus annuus* seed oil” is the most used sunflower preparation in both years, but in 2018, “*Helianthus annuus* seed wax” (a product from sunflower oil winterization) was also documented. Aside from its moisturizing properties, no evidence from the interest of sunflower in anti-aging cosmetics was found.

#### 2.2.6. *Theobroma cacao*

*Theobroma cacao* (cocoa) is part of the Sterculiaceae family, and its nutritional and medicinal purposes are documented since the Mayan and Aztec civilizations [61]. 

Cocoa beans, which are the raw material of cocoa butter, contain approximately 50% of lipids. The lipid fraction contains mainly triglyceride molecules such as palmitic acid, stearic acid, oleic acid, and linoleic acid [32,62]. Cocoa beans also contain a relevant polyphenol concentration, which is why in 2012, cocoa powder was considered one of their greatest sources by the European Food Safety Authority [63]. Flavonoids are the main polyphenols from cocoa beans, including catechin, epicatechin, procyanidin B1, procyanidin B2, and procyanidin C1 [64]. Xanthine derivatives, caffeine, and theobromine are also important components from cocoa beans, and they contribute to their antioxidant action [65]. The alkaloid theobromine has shown to prevent photodamage in hairless mice exposed to solar-simulated ultraviolet irradiation, by reducing wrinkles formation, dermal connective tissue alteration, and collagen accumulation. One study also suggested that xanthine derivatives prevent neutrophil infiltration caused by UV irradiation, supporting a critical role for theobromine in the dermal protective action exhibited by cocoa [61]. The sparse use of “*Theobroma cacao* seed butter” in 2011, possibly as emollient, has ceased in 2018. However, “*Theobroma cacao* (cocoa) seed extract” started to appear in anti-aging products during the later year, which was possibly due to its recent application against the blue light effect. This ingredient contains cocoa peptides, which were shown in in vitro studies to act during blue light stress by decreasing reactive oxygen species and maintaining opsin photoreceptors, while increasing collagen I, fibrillin-1, and syndecan-4 [66].

#### 2.2.7. *Calendula officinalis*

*Calendula officinalis,* also known as marigold, belongs to the Compositae family. The flower extract is rich in active compounds including terpenoids, carotenoids, flavonoids (the flavonols quercetin, rutin, narcissin, isorhamnetin, kaempferol), and volatile oils [67]. 

The antioxidant and antimicrobial activities from a “*Calendula officinalis* extract” in vitro have been demonstrated [68,69,70].

A single blind study with placebo control was performed using an emulsion containing marigold extract applied over a period of 8 weeks. This formulation showed the ability of inducing skin tightness (also known as skin firming or skin lifting effect), while increasing its hydration. However, it did not present a significant effect on skin elasticity [71]. The same group performed another single blinded placebo-controlled study that showed that the formulation containing marigold extract was able to decrease skin erythema and reduce transepidermal water loss (TEWL), which is a parameter that is linked to skin barrier function [72]. For this reason, marigold extract has been proposed as an anti-pollution ingredient [73].

Some of these studies do not specify which part of the plant was used to prepare the extract, although the evidence suggests the flower extract is the most commonly used for cosmetic application [67,74]. Similar to several other members of the Compositae family, marigold is known to cause allergic contact dermatitis, which may be a limitation for its use in cosmetic products [75].

Although the use of “*Calendula officinalis* flower extract” increased from 2011 to 2018 in anti-aging products, its efficacy in this regard remains unknown.

#### 2.2.8. *Limnanthes alba*

*Limnanthes alba* (meadowfoam) is abundant in the US states of Oregon and California, and it belongs to the Limnanthaceae family [76]. Its seed oil is richer in monounsaturated fatty acids than olive oil, being also a rich source of omega-6 and omega-3 fatty acids. In fact, meadowfoam is known to be the richest botanical source of docosahexaenoic acid (DHA) [15]. DHA is a key precursor of acetylcholine, being proposed to activate contractile fibers and increase skin firmness [77]. This oil has a significant antioxidant action, which is attributed to its fatty acid profile but also to its tocopherol content [78]. More recently, an in vitro study showed that two meadowfoam glucosinolate derivatives found in seedmeal (a waste product from the oilseed industry) are able to ameliorate UVB-induced DNA damage, modulate proliferation, and inhibit MMP expression in the skin microenvironment [76]. One split-face, prospective, double-blinded, controlled clinical trial was conducted with a cosmetic containing meadowfoam and onion preparations. After 12 weeks, there was a very significant reduction in tactile roughness, skin clarity, fine lines and wrinkles. However, the fact that this product contained several botanical preparations does not allow drawing any conclusions about the role of meadowfoam extract [79].

“*Limnanthes alba* (meadowfoam) seed oil” use has grown in 2018. However, the existing evidence is not enough to establish its efficacy as an anti-aging ingredient.

#### 2.2.9. *Glycyrrhiza glabra*

*Glycyrrhiza glabra* is often described as a medicinal plant, and it is originally from the former Mesopotamia in Middle East [80]. In 2011, only products containing the extract obtained from leaves were found. Plant leaves contain carotenoids, such as lutein and β-carotene, but also dihydrostilbenes, which were first found in this plant, and flavonoids, such as glabranin, licoflavanone, pinocembrin, and wighteone [81]. Both water-holding and permeability barrier function in the stratum corneum (SC) are essential for keeping skin moisture. Intercellular lipids in stratum corneum, which are composed mainly of cholesterol, fatty acids, and ceramides, play a crucial role in maintaining the function of SC. One study showed that licorice leaf extract not only promoted an increased hyaluronan production of mRNA expression levels of serine palmytoyl transferase and sphingomyelinase in vitro, which are involved in ceramide biosynthesis from keratinocytes, but it also promoted the syntheses of ceramides both in a 3D skin model and in human skin. Furthermore, licorice leaf extract increased the expression of mRNA for 3-hidroxi-3-methyl-glutaril-coenzime A reductase, which is related to cholesterol biosynthesis—an essential component of the skin barrier. Together, these effects are proposed to reduce the dehydration that is characteristic for skin aging [82].

Contrary to 2011, in 2018, only cosmetic products containing root extract were found. The ethanol preparation from licorice showed a high antioxidant activity by ROS scavenging, hydrogen-donating, metal ion chelating, mitochondrial antilipid peroxidative and reducing abilities. This was attributed to its high content in phenolic components, from which we highlight glycyrrhizin. Glycyrrhizin, the main constituent of licorice, is a glycyrrhetinic acid conjugate with two molecules of glucuronic acid [14]. Glycyrrhetinic acid, also known as enoxolone, is commonly used as a soothing agent in cosmetic products [83].

However, the isoflavonoid glabridin is the main compound from the hydrophobic fraction of licorice extract, and it has been shown to scavenge ROS and inhibit UVB-induced tyrosinase both in vitro and in vivo without affecting DNA synthesis [84,85]. Nonetheless, the reduced skin penetration and instability constrains the application of glabridin in cosmetic products [86]. Two clinical studies were found regarding cosmetic products containing glabridin in their composition. However, since glabridin was not the only active ingredient in these products, it is impossible to draw any conclusion regarding its effectiveness [87,88]. There are also flavonoids in licorice extract that act on tyrosinase inhibition, such as isoliquiritigenin, licuraside, isoliquiritin, and licochalcone A [14]. Liquirtin, on the other hand is a flavonoid that does not act on tyrosinase but causes depigmentation mainly by dispersing melanin. One study showed that the topical application of cream containing 2% and 4% liquirtin for four weeks was effective for the treatment of melasma, although both creams had a significantly lower efficacy than hydroquinone [89]. 

Overall, licorice extract was considered the depigmenting ingredient with the fewest side effects [90]. There is one clinical study including 12 individuals that tested the skin-lightening effect from the application of three creams containing 10, 20, and 40% licorice extract in different testing areas. There was a significant decrease in skin pigmentation after the use of all formulations, but their effectiveness was not significantly different with increasing concentrations [91]. Another small study evaluated the effects of a formulation containing 1% licorice extract in skin melanin and skin erythema. Licorice extract significantly affected skin melanin content but not skin erythema, comparing with placebo [92].

Considering the evolution from the use of licorice plant in cosmetic products, it seems that it has decreased from 2011 to 2018, even though the industry is moving toward the use of the plant extract that presents the soundest scientific evidence, namely *Glycyrrhiza glabra* root extract.

#### 2.2.10. *Acacia decurrens*

*Acacia decurrens* (black wattle) belongs to the Fabaceae family [93]. The lipids from the flower extract are the most used preparation in cosmetic products, providing a skin-conditioning and occlusive action [94]. “*Acacia decurrens* flower wax”, which only was found in cosmetic formulations from 2018, contains an association of free fatty alcohols (7%), saturated monoesters (22%), and a large amount of odd-numbered hydrocarbon chains (27%, C27-C33) [94,95]. No data connecting this preparation to an anti-aging benefit were found.

**Table 3:** Polyphenol composition of the top 10 botanical preparations, and their benefits for skin health.

Botanical Ingredient	Polyphenol Composition	Benefits for Skin Health	References
***Vitis vinifera***			
*Vitis vinifera* (grape) seed oil	Unknown	-	-
*Vitis vinifera* (grape) fruit extract	Anthocyanins	Antioxidant, UV-induced skin damage prevention	[96]
	Flavonols (quercetin)	Antioxidant, cell longevity increase	[97]
	Flavan-3-ols (epicatechins and catechins)	Antioxidant, UV-induced skin damage prevention, collagen synthesis activation, MMP inhibition	[98]
	Tannins	Antioxidant, astringent, wound-healing promotion	[99]
	Hydroxycinnamic acid derivatives	Antioxidant, UV-induced skin damage prevention, MMP inhibition, anti-inflammatory, anti-tyrosinase	[100]
	Stilbenes (resveratrol)	Antioxidant, UV-induced skin damage prevention, MMP inhibition	[24]
Palmitoyl grape seed oil	Unknown	-	-
Palmitoyl grapevine shoot extract	Unknown	-	-
Palmitoyl grape seed extract	Unknown	-	-
*Vitis vinifera* extract	Unknown	-	-
*Vitis vinifera* seed extract	Flavan-3-ols (epicatechins and catechins)	Antioxidant, UV-induced skin damage prevention, collagen synthesis activation, MMP inhibition	[98]
	Proanthocyanidins (procyanidin B1)	Antioxidant, UV-induced skin damage prevention, MMP inhibition, anti-inflammatory	[101]
	Stilbenes (resveratrol)	Antioxidant, UV-induced skin damage prevention, MMP inhibition	[24]
	Hyrdoxybenzoic acid derivatives (gallic acid)	Antioxidant, UV-induced skin damage prevention MMP inhibition, anti-inflammatory, anti-tyrosinase	[100]
***Butyrospermum parkii***			
*Butyrospermum parkii* (shea) butter extract	Unknown		
*Butyrospermum parkii* (shea) butter	Flavan-3-ols (catechin)	Antioxidant, UV-induced skin damage prevention, collagen synthesis activation, MMP inhibition	[98]
***Glycine soja***			
*Glycine soja* (soybean) oil	Unknown	-	-
*Glycine soja* (soybean) germ extract	Unknown	-	-
Hydrolyzed soy protein	Not applicable	-	-
Soy isoflavones	Isoflavones	Antioxidant, UV-induced skin damage prevention, anti-inflammatory, and estrogenic effects	[15,51]
***Simmondsia chinensis***			
*Simmondsia chinensis* (jojoba) seed oil	Tannins	Antioxidant, astringent, wound-healing promotion	[99]
*Simmondsia chinensis* oil	Tannins	Antioxidant, astringent, wound-healing promotion	[99]
Jojoba esters	Not applicable	-	-
***Helianthus annuus***			
*Helianthus annuus* seed oil	Hydroxycinnamic acid derivatives (chlorogenic, acid, caffeic acid, ferulic acid)	Antioxidant, UV-induced skin damage prevention, MMP inhibition, anti-inflammatory, anti-tyrosinase	[100]
*Helianthus annuus* seed wax	Unknown	-	-
***Theobroma cacao***			
*Theobroma cacao* seed butter	Flavan-3-ols (epicatechins and catechins)	Antioxidant, UV-induced skin damage prevention, collagen synthesis activation, MMP inhibition	[98]
	Proanthocyanidins	Antioxidant, UV-induced skin damage prevention, MMP inhibition, anti-inflammatory	[101]
*Theobroma cacao* (cocoa) seed extract	Unknown	-	-
***Calendula officinalis***			
*Calendula officinalis* flower extract	Flavonols (quercetin, rutin, narcissin, isorhamnetin, kaempferol)	Antioxidant, cell longevity increase	[97]
***Limnanthes alba***			
*Limnanthes alba* (meadowfoam) seed oil	Unknown	-	-
*Limnathes alba* (meadowfoam)	Unknown	-	-
***Glycyrrhiza glabra***			
*Glycyrrhiza glabra* (licorice) leaf extract	Dihydrostilbenes	Antioxidant	[101]
	Dihydroxyflavanones (glabranin, licoflavanone)	Antioxidant, anti-cancer	[102]
	Flavanones (pinocembrin)	Antioxidant,	[103]
	Isoflavones (wighteone)	Antioxidant, UV-induced skin damage prevention, anti-inflammatory, and estrogenic effects	[15,51]
*Glycyrrhiza glabra* root extract	Unknown	-	-
***Acacia decurrens***			
*Acacia decurrens* flower wax	Unknown	-	-

### 2.3. Botanicals’ Anti-Aging Mechanisms

**Figure 2:**
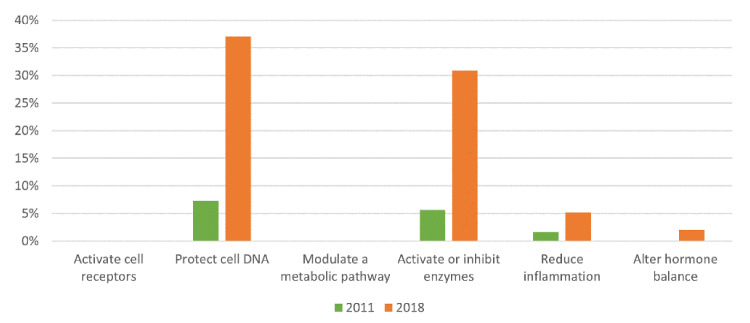
Relative amount of botanical ingredients classified by anti-aging mechanism.

After analyzing the literature regarding each botanical preparation, the ingredients whose anti-aging mechanisms are documented in the scientific literature were categorized and quantified (Figure 2).

Overall, there was an increase in the use of botanicals with proven anti-aging action from 2011 to 2018. It is noticeable that the most used categories are the same in both years. Botanicals with DNA-protecting action were used more frequently, followed by enzyme-inhibiting ingredients and those with inflammation-reducing action. It is also worth mentioning the emergence of ingredients that alter hormone balance.

The use of botanical ingredients that activate cell receptors and modulate a metabolic pathway appears to be less relevant. Comparing with a similar study based on the American market in 2010, only “*Vitis vinifera* grape seed extract” and “*Glycyrrhiza glabra* root extract” were mentioned in both studies [13]. 

Evidence for the efficacy and mechanism of action from botanical ingredients is sparse, and it often lacks relevance. In addition, the multitude of factors affecting botanical preparations composition, from plantation to the extraction method, adds an even greater complexity to this discussion. It is not easy for formulators to choose the most appropriate botanical ingredients for the development of cosmetic products, which ultimately may impact a product’s performance. This is not only detrimental for the consumer but also for the manufacturer, which may find it harder to generate and substantiate appealing claims.

## 3. Materials and Methods

### 3.1. Data Collection

Data were collected from anti-aging cosmetic products from multination brands marketed in pharmacies and parapharmacies in Portugal. Anti-aging cosmetics were included in the study if they exhibited in the label one of the following words: anti-wrinkle(s); anti-age/anti-aging; wrinkles repair; regenerator; aging; firming. All the information available in the product’s label was collected, along with the information available on the manufacturers’ websites. The data collection started in 2011 and was updated with products launched in 2018 or whose composition has been renewed that year, in order to avoid duplicate product analysis and to reflect the market trends. Cosmetics for application on the face, neck, and eye contour were included, comprising more than 40 multinational brands. Following these criteria, 280 products were selected, namely 177 and 103 respectively in 2011 and 2018.

### 3.2. Botanical Preparations Prevalence and Variety

The relative amount of cosmetic products containing anti-aging botanical preparations (expressed in percentage) and the variety of preparations used for each year were determined.

### 3.3. Top Botanical Species

Each botanical preparation was listed according to its INCI name and then classified according to the botanical’s scientific name. The analysis focused on the ten botanical species (Plantae kingdom) with highest usage frequency among the selected products. The usage frequency for each botanical species was determined by the sum of the number of products containing that specific preparation in both years and then ranked in descending order.

### 3.4. Evidence for Botanicals Anti-Aging Efficacy

The preparations’ composition and respective anti-aging benefits were searched on the following online databases: PubMed, Google Scholar, Scopus, Cochrane, and KOSMET. Then, the botanical preparations that showed anti-aging activity in clinical trials were grouped according to their mechanisms of action, as described by Hyland Cronin and Zoe Draelos [13]. This classification system categorizes the anti-aging ingredients according to eight distinct mechanisms of action: “modify the skin barrier” (e.g., smooth skin scale, exfoliate skin scale), “enhance intercellular lipids” (e.g., cholesterol, triglycerides, essential fatty acids, ceramides, natural moisturizing factor), “activate cell receptors” (e.g., retinoids), “protect cell DNA” (e.g., antioxidants, sunscreens), “modulate a metabolic pathway” (e.g., peptides), “activate or inhibit enzymes” (e.g., skin-lightening agents), “reduce inflammation” (e.g., botanic antioxidants, plant sterols), or “alter hormone balance” (soy phytoestrogens). Botanicals that were found to solely modify the skin barrier and/or enhance intercellular lipids were not considered as anti-aging active ingredients. Those categories are not specific from the anti-aging segment, which is why these ingredients may be used at a given formulation because of their occlusive/emollient or viscosity modifying actions. Therefore, their inclusion as anti-aging active ingredients could bias this study.

## 4. Conclusions

The cosmetic industry is always evolving, and the competition together with the need to meet consumers’ preferences require constant product development and reformulation. Nature-derived ingredients are increasingly popular. The prevalence of botanical preparations in the composition of anti-aging products has increased from 2011 to 2018. During this period, *Vitis vinifera*, *Butyrospermum parkii*, and *Glycine soja* have remained in the pole positions. These three plant species, together with *Theobroma cacao* and *Glycyrrhiza glabra*, have shown to be effective as anti-aging active ingredients. These botanical preparations stand out for their content in compounds with interest for anti-aging cosmetics, and their efficacy has been shown both in vitro and in vivo. It is noteworthy that all these preparations contain polyphenols, mostly flavonoids, but also stilbenes. Among the various flavonoid families, flavan-3-ols were the most frequently found in the top 10 botanical preparations, followed by proanthocyanidins, anthocyanins, flavonols, isoflavones, and tannins. On the other hand, botanical species such as *Simmondsia chinensis*, Helianthus *annuus*, *Calendula officinalis*, *Limnanthes alba*, and *Acacia decurrens* have insufficient or non-existing data supporting their use as anti-aging actives, and they are likely to be incorporated in formulations for their emollient or rheological-modifying properties.

Regarding the function of the botanical preparations studied, it is clear there is a preference for DNA-protecting ingredients, followed by ingredients that are able to activate or inactivate enzymes and inflammation-reducing ingredients. It is noteworthy that ingredients that alter hormone imbalance have reached the top 10 only in 2018, which may predict a trend for the coming years. In conclusion, despite the widespread use of botanical ingredients in anti-aging cosmetics, only a few preparations were studied for their anti-aging effectiveness, mainly using in vitro tests. In vivo randomized controlled trials are needed to strengthen the evidence for the use of botanicals as anti-aging active ingredients.

## 5. Limitations

Over the period of seven years, the noticed changes in the use of botanical preparations could have been influenced by the methodology used, since data collection in 2018 has focused only on new and renewed formulations, which might have resulted in the study of different brands across the years.

In addition, botanical preparations’ properties are related to a wide variety of environmental factors and maturation degrees, as well as the extraction methods. Even though some botanicals possessed a well-documented anti-aging activity, the efficacy of the final product also depends on the formulation. Several variables can greatly affect the efficacy of formulations containing botanical active ingredients such as their concentration and release, the presence of penetration enhancers, or special skin delivery systems, but also botanical preparations stability and their interaction with other ingredients of the formulation.

## Figures and Tables

**Table 2:** Botanical ingredients found in INCI lists from analyzed anti-aging products and their relative usage.

Botanical Ingredient	2011 (%)	2018 (%)
***Vitis vinifera***		
*Vitis vinifera* (grape) seed oil	3.95	2.91
*Vitis vinifera* (grape) fruit extract	1.69	0.00
Palmitoyl grape seed oil	0.56	0.00
Palmitoyl grapevine shoot extract	6.21	3.88
Palmitoyl grape seed extract	0.00	3.88
*Vitis vinifera* extract	0.00	0.97
*Vitis vinifera* seed extract	0.00	0.97
***Butyrospermum parkii***		
*Butyrospermum parkii* (shea) butter extract	0.00	0.97
*Butyrospermum parkii* (shea) butter	5.65	14.56
***Glycine soja***		
*Glycine soja* (soybean) oil	1.13	6.80
*Glycine soja* (soybean) germ extract	0.00	3.88
Hydrolized soy protein	3.39	0.00
Soy isoflavones	0.00	0.97
***Simmondsia chinensis***		
*Simmondsia chinensis* (jojoba) seed oil	0.56	0.00
*Simmondsia chinensis* oil	0.00	4.85
Jojoba esters	0.00	6.80
***Helianthus annuus***		
*Helianthus annuus* seed oil	1.13	8.74
*Helianthus annuus* seed wax	0.00	0.97
***Theobroma cacao***		
*Theobroma cacao* seed butter	0.56	0.00
*Theobroma cacao* (cocoa) seed extract	0.00	6.80
***Calendula officinalis***		
*Calendula officinalis* flower extract	1.69	4.85
***Limnanthes alba***		
*Limnanthes alba* (meadowfoam) seed oil	0.56	4.85
*Limnathes alba* (meadowfoam)	0.56	0.00
***Glycyrrhiza glabra***		
*Glycyrrhiza glabra* (licorice) leaf extract	3.39	0
*Glycyrrhiza glabra* root extract	0	1.94
***Acacia decurrens***		
*Acacia decurrens* flower wax	0.00	6.80

## Data Availability

The data presented in this study are available on request from the corresponding author. The data are not publicly available due to privacy.
